# “The days are long but the decades are short”: subjective aging, life trajectories, and incel identity formation among self-identified older incels

**DOI:** 10.3389/fpsyg.2026.1948952

**Published:** 2026-10-01

**Authors:** Linda Coufal, Viivi Korpela

**Affiliations:** 1 Institute of Sociology, Faculty of Social Sciences, Charles University, Prague, Czechia; 2 Department of Social Sciences and Philosophy, Faculty of Humanities and Social Sciences, University of Jyväskylä, Jyväskylä, Finland

**Keywords:** age-salience, incels, life trajectories, misogynistic extremism, misogyny, subjective aging, older adults

## Abstract

Misogynistic radicalization and incel identity formation are often discussed as problems of young men. However, the hub of online misogynistic discourse, the manosphere, has always been aimed at men of all age groups. Research tends to omit older users in the analysis, even though they are active members of extremist communities. This article examines how self-identified older incels narrate their life trajectories and explain the processes through which they came to adopt, maintain, intensify, or question an incel identity. We ask: (1) How do older incels narrate life trajectories to explain how they became and remain incels over time?, and (2) What role do subjective experiences of aging (a process of becoming older) play in incel identity formation? The analysis is based on 19,453 publicly accessible posts from incels.is written by 43 users whose usernames made age, aging, or generational position salient. Through inductive thematic analysis, we show that older incels narrate inceldom not as a sudden ideological shift, but as the cumulative outcome of early exclusion, missed romantic and sexual milestones, social withdrawal, failed self-improvement, and perceived trajectory lock-in. Aging becomes a key meaning-making resource: specific ages are constructed as deadlines, bodily changes as evidence of sexual disqualification, and accumulated time as proof that inceldom has become permanent. However, aging does not produce a single emotional or ideological trajectory. While some forum users express resentment and violent fantasies, others describe exhaustion, numbness, resignation, or ambivalent attempts to advise younger incels. The article contributes to incel and radicalization research by showing how harmful online identities may be sustained and emotionally reorganized across the life course.

## 1 Introduction

Misogynistic radicalization is considered to be mainly a problem of young men. It is a phenomenon distinctly rooted in social media culture, a problem highlighted by high school and middle school teachers (Roberts and Wescott, 2024), and research has indicated that large percentages of users on these forums are under 25 (Jaki et al., 2019). It is also predominantly socially isolated young men who end up radicalized to violence (Bizina and Gray, 2014). However, the hub of online misogynistic discourse, the manosphere, has always been aimed at men of all age groups, as evidenced by groups such as Wise Old Men (WOM) and Old Married Guys (OMGs) (Tomkinson et al., 2020). Researchers have so far largely ignored the existence of older individuals within this misogynistic online ecosystem and within extremist movements in general, even though younger and older radicalized people differ socially and psychologically (Oppetit et al., 2019). Furthermore, as Halilovic Pastuovic and Wylie (2025, p. 74) point out, “*too much focus on youth as a driver of extremism misses the existence of older participants in extremist violence, [and] clouds understanding of the reasons for their violent acts*”.

Within the manosphere, incels (involuntary celibates) are the only manosphere group to which terrorism has been linked (Lounela and Murphy, 2024; Tomkinson et al., 2020). Incels are also claimed to hold the most radical ideology in the manosphere (Ging, 2019) and have been studied as a representative case of misogynist extremism (O’Hanlon et al., 2024). Incels self-define in relation to the absence of romantic relationships (Cottee, 2021), which they see not only as a source of personal frustration, but a moral wrongdoing inflicted upon them broadly by society as a whole, and mainly by women. They politically mobilize around this relationship deficit and develop a complex ideology that explains how society is structured into hierarchies of sex and attractiveness (Baele et al., 2021), which they call the black pill ideology. Researchers have studied this ideology as misogynist extremism (Coufal et al., 2025; Coufal and Wedel, 2025; O’Hanlon et al., 2024; Tomkinson et al., 2020) or the black pill radicalization pipeline (Green et al., 2023). Not all incels are, however, radical and misogynistic (Kelly et al., 2022), and the “*prevailing condition among incels is one of apathy, not violent rebellion*” (Cottee, 2021, p. 108).

Yet, a systematic review of the incel research highlights that the ideology is violent on three levels: societal, interpersonal, and personal, suggesting that not just violent misogyny, but also self-harm is one of the violent avenues of incel ideology (Aiolfi et al., 2024). Mental health struggles have also been found to be one of the most important topics on the forum (Wedel and Coufal, 2025). Researchers have pointed to depression, anxiety, feelings of loneliness, and suicide as some of the factors that may lead to a person self-identifying as an incel (Aiolfi et al., 2024, p. 26272). Therefore, despite the forum often serving as a mental health outlet for its users, it has been found that it often encourages self-hate and self-harm.

In this paper, we study self-identified older incels. By focusing on older incels, we shift the analysis from incel identity as a moment of ideological attachment to incel identity as a life-course formation. The retrospective accounts written by older incels make visible how early exclusion, missed romantic and sexual milestones, social withdrawal, and aging are narrated as cumulative rather than isolated experiences that together form incel identity. Older incels’ accounts therefore allow us to examine not only why individuals may enter incel spaces, but also how incel identity is maintained, intensified, softened, or stabilized over time. In this sense, older incels are analytically significant not because they represent all incels, but because they show how time itself becomes part of incel meaning-making. This temporal perspective is particularly relevant because incels are an online community characterized by loneliness and social isolation (Aiolfi et al., 2024), both of which are recognized concerns also in later life (Luhmann and Hawkley, 2016) due to, among other factors, shrinking social networks, living alone, widowhood, declining health, and retirement.

Within incel ideology, there is a broad range of incel identities corresponding to various reasons for a person’s involuntary celibacy. Among these is oldcel, referring to old age as the reason for one’s lack of romantic success. The existence of these users has been neglected in the research on incels so far. However, we argue that their perspective is crucial for understanding not only the social, cultural and affective factors, but also the life and emotional trajectories that influence identity formation. The name “involuntary celibate” already signals the centrality of romantic and sexual exclusion to incel identity. For older users, however, this exclusion is not only a present condition, but something that can be narrated as having accumulated across decades. As Westerhof and Wurm (2015) state, humans are able to reflect on who they are and how they are growing older. Therefore, investigating how older incels explain life trajectories that led them to adopt an incel identity is uniquely suited not only to uncover the factors leading to the development and maintenance of incel identity, but also to see what may intensify or weaken it. Furthermore, people make sense of who they are by integrating reconstructed past experiences and imagined futures into an evolving life story (McAdams and McLean, 2013), and later life can thus involve retrospective evaluation, in which earlier experiences are revisited and given meaning in relation to the present (Butler, 1963).

In this article, we investigate how self-identified older incels narrate their life trajectories and explain the processes through which they came to adopt, maintain, or intensify an incel identity. Focusing on forum users’ retrospective accounts, we identify the social, cultural, and affective factors users articulate as central to their meaning-making and self-positioning within the incel worldview. Thus, we ask: (1) How do older incels narrate life trajectories to explain how they became and remain incels over time? and (2) What role do subjective experiences of aging (a process of becoming older) play in incel identity formation?

## 2 The appeal of incel ideology

Incels are a group of men on the internet who politically mobilize around their inability to find a sexual and/or romantic partner (Coufal and Wedel, 2025). Instead of interpreting this as a private source of frustration, they transform this circumstance into a larger political grievance and develop a broad theory of how the world functions, which many scholars argue is extremist (Coufal et al., 2025; O’Hanlon et al., 2024; Perliger et al., 2022). Incels are part of the manosphere, which is an ecosystem of online communities and influencers loosely connected around the idea that boys and men are victimized in contemporary society by female liberation–what they call The Red Pill theory. Incels differ from the rest of the manosphere by developing so-called Black Pill theory, which postulates that the personal situation imposed on the individual by society, as understood through The Red Pill, cannot be improved. This nihilistic angle is, according to many researchers, what creates the background for violent extremism perpetrated by members of the incel community (Green et al., 2023; Tomkinson et al., 2020). However, incel communities are not ideologically or socially homogeneous, and users vary in how strongly they invest in redpill, blackpill, self-harming, or extremist framings.

The incel online community is infamous for its online misogyny, terrorist attacks, and pioneering of hateful and violent language. However, apart from promoting interpersonal and societal violence, incel ideology is also filled with self-hate and violence towards oneself (Aiolfi et al., 2024). Perhaps not surprisingly, then, incels report “*high levels of depression (91%), anxiety (85%), Post-Traumatic Stress Disorder (40%), and autism spectrum disorders (53%). Almost all participants reported a history of being bullied (91%)*” (Moskalenko et al., 2022, p. 2). It is impossible to distinguish whether the self-hate aspect of incel ideology draws in individuals struggling with mental health, or whether the ideology developed this aspect as a response to members struggling with mental health and low self-worth. Regardless, bullying and loneliness seem to be a unifying experience of members of incel communities, often being discussed on the forums to seek relief and companionship under these difficult circumstances (Pettersson et al., 2025; Regehr, 2022; Tietjen and Tirkkonen, 2023).

However, through participation on the forum, loneliness is not alleviated but rather politicized and exacerbated. Central to the politicization of loneliness is the affective mechanism of ressentiment *“that transforms their loneliness into misogynistic antagonistic emotions”* (Tietjen and Tirkkonen, 2023, p. 1237). As a result, self-identification with the incel community rather deepens social isolation and intensifies feelings of low self-worth (ibid), and also deepens the feeling of victimhood, to the extent that 51% of respondents (Moskalenko et al., 2022, p. 2) reported being socially persecuted for their inceldom. Victimhood and absolution from individual responsibility are an integral part of the incel ideology. Incels are preoccupied with pseudoscientific explanations of their lack of romantic success (Cannito and Ferrero Camoletto, 2022). The most famous of these theories is the theory of “lookism”–discrimination due to one’s physical appearance (Baselice, 2024), which spread beyond the ecosystem of the manosphere into the mainstream (Solea and Sugiura, 2025). In the logic of The Red Pill theory, society operates around a so-called “sexual market,” and a person has a value on this market that depends on their socioeconomic status and physical attractiveness; what they call the Look, Money, Status theory. To achieve success in this sexual market, men need to have a high look-money-status “score,” which may be increased by behaving in a “patriarchal” masculine way, or through the praxis of looksmaxxing (Botto and Gottzén, 2024). Looksmaxxing is, at the time of writing, a viral praxis of enhancing one’s physical appearance through various means of differing invasiveness and extremity, that originated in the manosphere and *“cloak misogyny and scientific racism under the guise of self-improvement*” (Solea and Sugiura, 2025, p. 1).

The Look, Money, Status theory of the sexual market provides “a (pseudo)scientific basis for the definition of the incels themselves” (Cannito and Ferrero Camoletto, 2022, p. 602). Incels are those men who “score” the lowest in the looks part – in other words, men who are the ugliest. From the perspective of lookism, relational rejection is based on looks only and *“something entirely out of one’s control and thus, engagement with the real world quickly becomes a pointless exercise”* (Regehr, 2022, p. 146). From the incel perspective, lookism absolves individuals of personal responsibility, which can be both “seductive and addictive” (ibid). However, there is inter-community dialogue about the implications of this “social reality.” Within the incel community, ideological divisions exist regarding the most influential factor in sexual interactions. While one faction views physical attractiveness as the most important factor – further splitting into those who believe appearance can be enhanced and those who view it as immutable – another faction postulates that a lack of physical beauty can be offset by financial wealth and high social status (Cannito and Ferrero Camoletto, 2022). This tension underpins internal debates surrounding the purity of incel ideology and identity. These disputes created the terms “truecell,” someone whose incel identity is pure and legitimate, and “fakecell,” someone whose incel identity is impure or illegitimate (Pettersson et al., 2025). Researchers have also found subcultural hierarchies tied to the “pills” of incel ideology and policing of behavior and beliefs similar to those found in other social groups (Schlaerth et al., 2024). The purity of incel identity is defended through loneliness and romantic failure (being “KHHV”: kissless, hugless, handholdless virgins), which ultimately fosters a culture of competitive victimhood, allowing members to feel special about their lack of romantic success (Maryn et al., 2024).

Another reason why users often join incel forums is a perceived struggle to meet masculinity norms (Maryn et al., 2024). In fact, Eurocentric hegemonic masculinity and the cisgenderist, heteronormative gender order associated with it have been identified as one of the key mechanisms through which extremism on the forum functions (Aiolfi et al., 2024; Coufal et al., 2025; O’Hanlon et al., 2024). Therefore, users turn to the forum in search of support *“around shared experiences of bullying or marginalisation, both of which reinforced and further legitimated the blackpill worldview of inceldom as something that is ‘given’ and inevitable”* (Pettersson et al., 2025, p. 347). In this discourse, genuine struggles with societal norms and social belonging are transformed through the lens of patriarchal entitlement (Sugiura, 2021) and pseudoscientific theories of social order (Cannito and Ferrero Camoletto, 2022) into “wounded male victimhood” (Kelly et al., 2024). Wounded male victimhood is something intimately connected with violence (Díaz and Valji, 2019; Ferber and Kimmel, 2008), but engaging with this “toxic masculinity” content has also been found to heighten depression, lower self-esteem, and generally poor mental health among men (Parent et al., 2019).

## 3 Beyond youth: age-salience and subjective aging within extremist communities

Research on radicalization and misogynistic extremism often foregrounds youth. However, as Halilovic Pastuovic and Wylie (2025) argue, treating extremism primarily as a youth problem can obscure the presence of older participants and limit our understanding of how extremist identities are shaped across longer life trajectories. Their argument challenges the assumption that radicalization is primarily driven by young men’s attraction to violence (Crone, 2016), or that older people are necessarily protected from radicalization by family and community ties (Schirch, 2018). Rather, they suggest that radicalization *“is not a rapid and individualised process”* but may be embedded in longer social and biographical contexts (Halilovic Pastuovic and Wylie, 2025, p. 81). Similarly, research comparing radicalized minors and adults suggests that age matters for understanding the different social and psychological contexts through which radicalization unfolds (Oppetit et al., 2019).

Age matters from another perspective as well: it can shape not only how radicalization unfolds, but also how older adults’ participation in digital spaces is recognized and interpreted in general. Dominant public narratives and policy discourses often portray older adults as fragile, vulnerable, or technologically lagging (Kebede et al., 2021; Levy, 2009; Van Leeuwen et al., 2024). Yet older adults are not merely passive observers of digital culture, but active participants in digitally mediated life (Korpela et al., 2023), including harmful or radicalized online contexts (Wells, 2023). This view of older adults as active digital participants is consistent with previous research showing that older individuals purposefully negotiate digital participation, support, and belonging in everyday life (Hänninen et al., 2023; Korpela et al., 2026). Older incels can therefore be understood as digital actors whose online participation reveals how aging, loneliness, masculinity, and ideological identity may become entangled in harmful online communities.

In this article, aging is not only a chronological process, but also a social and affective experience shaped by how people interpret their bodies, biographies, relationships, and futures (Diehl et al., 2014). People may feel old, too late, or out of time before reaching what would conventionally be understood as old age. Accordingly, we approach age as a form of self-presentation that creates age-salient social contexts. By age-salience, we refer to moments when age, aging, or generational position is made visible and meaningful for identity and social positioning (Giles et al., 2010). Throughout adulthood, individuals navigate culturally scripted “social clocks,” referring to normative expectations surrounding the timing of partnership, family formation, career trajectory, and aging-related transitions (Havighurst, 1976). Falling outside these culturally scripted timelines can produce an acute sense of “off-time” aging.

This is particularly relevant in incel forum culture, where usernames such as “oldcel” do not simply indicate possible chronological age, but make aging itself part of the user’s incel identity. The focus of this paper is therefore not on verifying whether users are objectively old, but on examining how “oldness” is mobilized as a meaningful category through which users interpret their intensely misogynistic and often extremist incel identity. Accordingly, subjective aging, referring to how adults perceive and experience their own aging (Westerhof and Wurm, 2015), is understood as the lived and narrated experience of becoming older, especially in relation to missed intimacy, bodily change, social comparison, and perceived closure of opportunity.

Building on this understanding of age-salience and subjective aging, we also draw on retrospective evaluation and life review concepts (Butler, 1963) which shift attention from isolated moments of radicalization to accumulated experiences, transitions, and turning points, embedding extremist identity in personal biography over time. Rather than focusing solely on when or why someone enters an extremist community, retrospective evaluation and life review illuminate how earlier life experiences and transitions can be revisited and given meaning in relation to the present. Older users’ accounts provide a longer retrospective vantage point for this analysis. By examining how self-identified older incels narrate becoming and remaining incels over time, we aim to capture how incel identity is maintained not only through misogynistic ideology, but also through retrospective life stories in which exclusion accumulates, opportunities close, and aging becomes evidence that the self has become permanently fixed outside normative intimacy.

## 4 Methodology

We approached our research questions through analysis of the largest incel forum: incels.is. Incels.is is an online forum, the content of which is predominantly visible without a login. However, to post, one must make an account. The forum is organized into threads to which users can reply, or they can start a new thread. Additionally, users can reply to any post in a thread without starting a new thread (Coufal and Wedel, 2025, p. 4). The analysis is based on all posts visible on the forum without registration, in the period from November 8, 2017, when the forum was founded, to September 1, 2024, when the data collection was completed. This dataset consists of 11,000 individual users and 5,749,536 individual posts. For each post, we know the author, the time of posting, and the content of the post. Out of the 11,000 users, we identified 112 whose usernames included explicit references to age, using keyword filtering (keywords: gray, grey, boomer, old, senior, pensioner, retired, elder), similar to Němcová and Coufal (2026).

We manually reviewed these usernames and identified 48 usernames that could be interpreted as age-salient self-presentations. Usernames were coded as age-salient when they contained explicit age indicators; direct self-identification as “old” or “elder”; culturally recognized metaphors of aging, such as greybeard, bald, or silver; a numerical age of 25 or above; or references to nostalgia, generational identity, tradition, legacy, or endurance over time. Because greycel/graycel can also refer to forum status rather than age, usernames containing grey/gray were not treated as age-salient on that basis alone. Instead, they were retained only when manual review and first-round close reading showed that the term referred to aging-related self-presentation, such as oldness, balding, or grey-beard imagery. The threshold of 25 was used because previous research suggests that incel forums are largely populated by younger users, with substantial proportions under the age of 25 (Jaki et al., 2019). This approach does not verify users’ chronological age. Rather, it captures how age was made visible and meaningful through forum self-presentation. This is analytically appropriate for the present study because our focus is not on older adults as a demographic category, but on self-identified older incels and the role that age, aging, and perceived oldness play in incel identity formation. We then read the posts of these 48 users and excluded five whose posts were not relevant to the analysis or whose age-salient username did not correspond to meaningful age-related self-positioning in their posts. The final sample consisted of 43 users and 19,453 posts, comprising 711,552 words in total.

The data was collected as text files (.docx exports) and treated as naturally occurring online discourse. It was analyzed through six steps of inductive thematic analysis (Braun and Clarke, 2006), a flexible method for identifying and interpreting patterns of meaning across qualitative data. The analysis began with repeated close readings of the posts to become thoroughly familiar with the material. Open coding was conducted in ATLAS.ti to capture recurring patterns. Codes were collated into preliminary themes, which were then iteratively reviewed to ensure they formed coherent patterns within coded extracts and across the full dataset. Throughout, we moved back and forth between data, codes, and themes, revising the coding where needed to keep interpretations grounded in the original posts. Finally, we integrated analytic interpretation with selected illustrative excerpts to present the findings clearly and situate them in relation to prior research (Braun and Clarke, 2006). Special attention was paid to how self-identified older incels connected personal experiences to broader social, cultural, and affective explanations, treating these as emic meaning-making resources in identity formation. Given the sensitivity of the topic and the presence of hateful language and self-harm references, we use data anonymization practices in reporting. This refers to removing usernames, paraphrasing where appropriate, and limiting direct quotations to short extracts necessary for analytic transparency. The following section presents the results of the thematic analysis.

## 5 From exclusion to permanence: life trajectories and aging in incel identity formation

The results section unfolds as a life-course narrative focusing on how incels.is forum users themselves experienced and narrated becoming and remaining incels over time. First, we examine how self-identified older incels construct adolescence and early adulthood as decisive periods of social sorting, exclusion, and romantic failure, thereby addressing RQ1: How do older incels narrate life trajectories to explain how they became and remain incels over time? We then move on to explore how forum users describe later biographical ruptures, prolonged isolation, and accumulated disappointments as factors that reinforced and *locked in* these trajectories over time. Building on this, the second part of the analysis further investigates the role of subjective aging in incel identity formation (RQ2), showing how forum users mobilize “feeling old” and “premature oldness” as an affective and social category associated with exhaustion, missed opportunities, permanence, and resignation. Together, the findings illustrate how early exclusion, accumulated biographical harm, and perceived temporal closure became intertwined in the construction and maintenance of incel identity.

Across the data, self-identified older incels did not describe becoming incel as a sudden ideological shift. Instead, they looked back on their lives and presented childhood, adolescence, adulthood, and aging as connected stages in a longer trajectory toward inceldom. The incel label often came later, giving these trajectories a name and a logic: early humiliation became evidence of social sorting, later isolation became evidence of trajectory lock-in, and aging became evidence that the trajectory had become permanent. The following themes trace this movement from origin stories to permanence.

### 5.1 “Winners and losers are decided early”: narrating the origins of inceldom

Although narratives of adolescent exclusion are common in incel communities more broadly, we analyze them here as retrospective life-course narratives produced by self-identified older incels. Their significance lies not in adolescence as a unique topic, but in how older users return to adolescence from later life and reinterpret early exclusion as the beginning of a trajectory that has hardened over years or decades. In these accounts, youth is narrated through the lens of accumulated time.

Forum users repeatedly constructed adolescence as the moment when romantic and social hierarchies first became visible. These were not neutral recollections of youth, but retrospective origin stories that made later incel identity appear predictable from the beginning. School was commonly described as a sorting institution where some boys were selected as desirable, socially fluent, and future-oriented, while others were marked as permanently outside normative intimacy, as forum user U1 explains below:

*I remember reading an article that by jr. high or end of jr. high girls have already picked who will be the sex havers and non-sex havers. Basically, by then, girls have already chosen who the winners will be, with the rest of men fighting a never ending battle. (U1).*

This quotation shows how junior high becomes a decisive point in the user’s retrospective life story: a moment at which later romantic and social exclusion is imagined to have already been set in motion. While this account is specific, the broader pattern was echoed across the data, and adolescence was repeatedly narrated as the period when future romantic and social possibilities were already beginning to close. From an analytical perspective, this operates as both a retrospective life-course claim and an identity claim, presenting inceldom as the adult name for a status framed as having been assigned long before adulthood even began. While such origin narratives also appear among younger incels, they take on particular significance here because older users narrate them from the standpoint of accumulated adulthood.

Importantly, early sorting was not described as abstract or invisible. It was experienced through everyday peer interactions, especially humiliation and exclusion. Bullying was therefore one of the central mechanisms through which self-identified older incels explained this early sorting. Rather than treating bullying as a discrete childhood harm, forum users often positioned it as the social and emotional foundation for adult avoidance, low confidence, distrust, and perceived romantic impossibility. As the excerpt below shows, the continuity from kindergarten to high school gave exclusion a developmental shape: it was not remembered as a single event, but as an atmosphere that structured the self over time.

*I was severely bullied from senior kindergarten all the way through to senior high school. By both boys and girls. I was mocked, teased, had mean nick names chanted at me while the whole classroom pointed and laughed. Malicious lies were spread about me. I was surrounded and gang-beaten repeatedly. [.] In high school, I was surrounded by a group of 4 or 5 seniors on my way home from school who beat me violently, breaking my nose and cheek bone. (U2).*

This type of account also linked adolescence to gendered competence. The forum users commonly framed teenage romance and sexual experimentation as necessary developmental training that enables adult masculinity. Missing this critical phase was therefore narrated not only as deprivation, but as arrested development. As a result, the adult incel self was imagined as someone who had not acquired the embodied and social capacities that others learned on time.

*Teen years for men are incredibly important in learning to act sexually direct and dominant as a man. Miss it there and you’ll fumble long into your adult life. (U3).*

Together, these accounts show how self-identified older incels retrospectively narrate adolescence as the point where exclusion became developmental and durable. Early bullying, romantic non-recognition, and missed sexual learning are made meaningful through later aging: they are not presented as isolated youthful experiences, but as the beginning of a life trajectory that came to feel increasingly irreversible over time.

### 5.2 “After that, everything went downhill”: biographical ruptures and trajectory lock-in

The second theme focuses on the later events and accumulated conditions through which self-identified older incels described their trajectories as becoming *locked in*. These narratives moved beyond early romantic failure to include family trauma, mental health struggles, economic insecurity, long-term isolation, failed self-improvement, and withdrawal from social worlds. The key analytic point is that the forum users did not present inceldom as only a dating status. They embedded it in broader biographies of social disconnection and shrinking future possibility. Below, one forum user narrates a catastrophic rupture followed by retreat:

*I lost both my parents about 20 years ago. Mother shot and killed my father then killed herself. [.] I inherited a large sum of money and tried to shut myself off from the world as best I could. (U1).*

The significance of this account is not that trauma mechanically produces incel identity, but that trauma can become one of the resources through which the forum user explains adult isolation. The attempt to shut himself off from the world is presented as both an understandable response and a turning point that narrowed future forms of connection. Importantly, this narrative of permanent exclusion was not framed as passive withdrawal. Some users described substantial attempts to change their situation, including spending significant amounts of money on cosmetic procedures, fitness regimens and other forms of bodily self-improvement–strategies known within the manosphere as *looksmaxxing*. Yet these attempts were often folded back into the same narrative of inevitability: even when users tried to alter the conditions of exclusion, the outcome was interpreted as proof that nothing could fundamentally change. Forum user U4 echoes this experience, explaining that even active attempts to change one’s situation could be interpreted as evidence of permanence:

*The worst of what I’d imagined came true: no matter what I did, the results always ended up being the same. Nothing will ever change in the life of an incel, no matter how much he yearns for it. (U4).*

Here, lock-in was narrated through the exhaustion of agency: repeated effort did not open new possibilities, but confirmed the belief that the trajectory cannot be changed. This sense of permanence was further intensified by aging. As users moved further into adulthood, exclusion was no longer described only as personal failure or romantic rejection, but as a widening social condition. Peer networks were imagined as reorganizing around partners, children, and family life, leaving the older incels increasingly outside ordinary forms of belonging:

*The older I get the more I realize that women completely control society and particularly social life. If there’s no women in your life, then you’ll just be completely shut out once your friends have kids. Then it’s over. (U3).*

At this point, aging entered the lock-in narratives as a form of relational accumulation. Self-identified older incels described adulthood as a period in which peer networks reorganized around partners, children, and family life. For those outside these pathways, growing older meant losing access not only to romance, but also to ordinary social belonging. Aging as an incel was therefore narrated as the absence of intimacy and progressive exclusion from the adult social world: the longer one remained outside coupled adulthood, the more social life itself appeared to withdraw. In this context, online spaces became important sites for recognition and validation.

### 5.3 “It’s too late now”: subjective aging as closure and exhaustion

The third theme turns to how forum users made sense of aging itself. Rather than treating oldness as a purely chronological category, they described it as an affective and social condition marked by exhaustion, missed timing, and perceived closure. To feel old was to feel that the socially meaningful window for intimacy, self-repair, or transformation had closed. This was why self-identified older incels in their thirties, forties, fifties, and sixties often used similar language of exhaustion and finality even though their chronological ages differed. This sense of closure appeared in compressed temporal rules and in longer retrospective accounts:

*That makes me right around 40 years old, which is last call even for normies. (U3).*

*Obviously I know now. But this was all the way back 1985 when I was 16. It’s too late now. I’m 50 and I’m still incel. So there’s no point telling me anymore. I’m too old now. (U5).*

For some users, the window of possibility was imagined to close already in the twenties. For others, later age became proof that earlier missed opportunities could no longer be repaired. The problem is therefore not simply that a user is twenty-five, thirty, or fifty, but that specific ages were treated as deadlines after which advice, self-improvement, or romantic hope arrive too late. In this sense, feeling old means feeling temporally out of place: still desiring intimacy, but imagining that the socially meaningful time for achieving it has already passed. Alongside this language of deadlines, forum users also described aging as temporal dissonance: the feeling that time had moved forward while the self remained emotionally attached to adolescence.

*Most people who are young now do not know how fast time flies as you get older. Months merge into a year. Years merge into a decade. And before you know it decades just zoom by. The kick to the teeth is that you as a person do not feel any differently than you did as a teenager, you just look a shit ton worse. (U6).*

Forum user U6 captures this temporal dissonance particularly clearly. The self was experienced as continuous with adolescence, while the body and social position were experienced as aged. This mismatch intensified missed-life grief, and many felt that they had carried the same unmet desires across decades while the external conditions for fulfilling them had deteriorated. As a result, bodily changes such as balding, wrinkles, low energy, or declining sexual confidence were not described merely as signs of getting older. Rather, they were interpreted as evidence of social and sexual disqualification, or what some forum users called and perceived as the “expiration” of masculinity. In the end, many self-identified older incels experienced oldness prematurely: they felt aged out of romantic possibility before reaching what would normally be understood as old age.

*You’re in much better situation than me. Because I started going bald at age of 19. (U7).*

As articulated by forum user U7 above, balding at nineteen can become more than a bodily change; it marks the moment when youth is perceived to have ended early. This illustrates how subjective aging can begin long before chronological aging: the body becomes a sign that the future has already narrowed.

### 5.4 Aging, permanence, and incel identity

The final theme moves from feeling old to the identity consequences of aging. Given that forum users narrated inceldom across years and decades, time itself became evidence. The persistence of loneliness, rejection, or non-participation was used to confirm that inceldom was not temporary. For some, this intensified resentment and anger. For others, it produced exhaustion, apathy, or pragmatic resignation. These affective outcomes were not mutually exclusive. They could coexist within the same broader identity formation.

*Being 41. this hits hard, but it’s the truth. There is no turning back, it’s completely over. Just to remain shut in until our bio functions cease. You think you are tired and bitter in your 20s? you know nothing yet! (U8).*

Here, as forum user U8 explains, aging intensifies permanence. He positions younger incels as unable to understand the full weight of long-term inceldom because they have not yet accumulated enough time. Older age becomes a form of negative authority: the forum users often claimed to know what true inceldom actually is when it is no longer imagined as a temporary phase. This authority was especially visible in how self-identified older incels positioned themselves in relation to younger incels. Age allowed them to speak not only as sufferers, but as witnesses to what inceldom becomes over time. Their accounts often carried an implicit warning: younger incels might still imagine escape, while older incels knew the emotional and social consequences of remaining outside intimacy for decades.

At the same time, older users did not always use age-based authority solely to reinforce hopelessness. Some positioned themselves as advisers to younger incels, encouraging them to keep trying and not to treat early rejection as permanent. This complicates the role of aging in the forum: older users could appear as warnings of what inceldom becomes over time, but also as figures of support who insisted that younger users’ futures were not yet fully closed. In this account, age provides a retrospective perspective. The forum user U6 looks back on a younger self who lacked the community now available online:

*I see a lot posts from younger guys, but not so much the older crew. Are there not a lot of us around? I really wish something like this website existed when I was a lot younger. I think it would have helped not feeling alone in the world. (U6).*

Importantly, aging does not automatically produce escalating rage. Some described anger giving way to resignation, numbness, or a more pragmatic orientation toward survival. But this does not mean that incel identity weakened. Rather, aging could stabilize the identity by changing its emotional texture: hope became less available, but so did the energy required for constant rage. This is why subjective aging is central to the analysis. It adds another layer to incel identity formation and reorganizes how forum users understand the past, assess the future, and inhabit the present.

*In my 20’s I still had hopes and dreams of breaking out of inceldom. But once I hit 30 I realized its not going to happen. [.] Once I hit 40 it was like a whole new experience. [.] I was super angry in my 20’s. Very bitter. Very angry. In my 40’s I’m finding myself to be the exact opposite. I’m a lot more mellower. A lot more calmer. (U6).*

Although resignation and emotional exhaustion were more common than explicit calls for violence, some posts showed how accumulated grievance could also move into violent fantasy or radicalization-adjacent language. In these cases, “going [initials of a terrorist]” functioned as a recognizable forum shorthand through which users referred to spectacular retaliatory violence. Such references did not necessarily indicate concrete intent, and some users explicitly rejected harming others. However, the presence of such posts shows that permanence was sometimes narrated not only through numbness or withdrawal, but also through fantasies of revenge against the social world perceived to have excluded them.

*The bitch, high maintenance looking hole gave me the stinkeye you get as a male, deemed below as her. As she walked by I made sure to stick my elbow out and made real impact. I kept walking. I just hope the cunt hurt from that. I will be doing this more regularly. Gave me good feelings, these stuck up whores deserve. [.] Next step, [name of the terrorist omitted] style. (U9).*

However, the emotional consequences of aging can be ambivalent. Older forum users did not describe a single affective trajectory from loneliness to rage. Instead, resentment, grief, exhaustion, apathy, and resignation often coexisted. For some, accumulated years intensified bitterness. For others, the same accumulation made anger harder to sustain. This ambivalence is central to understanding older incel identity formation: aging could harden the belief that inceldom was permanent while simultaneously softening, flattening, or exhausting the emotional energy attached to it.

*I’m 50 + and I’m still coping. To be honest I do not know how I do it. I’m pretty numb by now and apathetic about everything. Life feels kind of like a never ending dream. (U10).*

This quotation shows permanence without active rage. The forum user remains within an incel self-understanding, but the dominant feeling is numbness rather than anger. Rather than imagining change, escape, or revenge, he describes coping as a state of emotional suspension: life continues, but without a strong sense of participation in the social world. This matters because it shows that aging can stabilize incel identity through resignation as much as through resentment.

Taken together, the findings show that older incels narrate identity formation through a movement from early exclusion to perceived permanence. Adolescence is remembered as the period when the self was sorted; later ruptures, failed attempts, and social withdrawal are used to explain why the trajectory became difficult to reverse; subjective aging gives these experiences a language of closure; and accumulated time transforms inceldom into a durable identity. Aging, then, does not simply accompany incel identity. It becomes one of the narrative mechanisms through which inceldom is made coherent, enduring, and emotionally consequential.

## 6 Discussion and conclusion

The findings of this study demonstrate that for incels who subjectively identify as older, inceldom is not merely a contemporary dating status or a sudden ideological shift; rather, it is often narrated as the cumulative outcome of a lifelong trajectory. In addressing the first research question–*how older incels narrate life trajectories to explain how they became and remain incels over time*–the data reveal that forum users retrospectively construct a cohesive biography in which childhood, adolescence, and adulthood are interconnected stages of social exclusion. These narratives frame inceldom as a mature label applied to a status of social and romantic marginalization that was assigned long before adulthood. Consistent with existing literature, bullying and loneliness emerge as unifying foundational experiences, which users discuss on forums to seek relief, validation, and companionship amid difficult circumstances (Pettersson et al., 2025; Regehr, 2022; Tietjen and Tirkkonen, 2023).

Extending research that shows how digital technologies can support social connection and meaningful engagement in later life (Korpela et al., 2023, 2024), our results show that online forums can also become spaces where experiences of exclusion and unmet social needs are intensified. A key contribution of this study is therefore to show how the blackpill becomes persuasive not only as an abstract ideology, but as a biographical explanation. In this sense, time itself became evidence. The longer exclusion persisted, the more the nihilistic angle of blackpill appeared to explain not only present rejection, but an entire biography imagined to have gone permanently off-course. It is precisely this deeply normalized nihilistic angle that can create the psychological and cultural foundation for violent extremism perpetrated by members of the incel community (Green et al., 2023; Tomkinson et al., 2020).

The biographical framing extends beyond romantic relationships into a broader feeling of “trajectory lock-in,” where the incel identity is embedded within larger histories of social disconnection, neurodivergence (e.g., autism and anxiety), and institutional exclusion. Forum users frequently narrate their adulthood through the exhaustion of agency; repeated efforts to improve their situation do not open new possibilities but instead reinforce the belief that their trajectory is unalterable. This “lock-in” often includes economic insecurity and unemployment, signaling a generalized difficulty in entering adult institutionalized life. As peer networks reorganize around normative relational structures like heterosexual marriage and family, the older incel experiences a loss of ordinary social belonging. Within this context of structural and interpersonal isolation, online forums cease to be merely ideological spaces; they become vital coping mechanisms providing the recognition and validation unavailable in the physical world. In this sense, older incels emerge as active digital actors whose online participation demonstrates how subjective aging, loneliness, and ideological identity become deeply entangled in a harmful online space. Thus, rather than understanding older individuals’ digital lives through the lenses of absence, vulnerability, or digital exclusion (Kebede et al., 2021; Van Leeuwen et al., 2024), our findings align with research showing that older adults actively negotiate digital participation and belonging in everyday life (Hänninen et al., 2023; Korpela et al., 2026), even if that belonging manifests within extremist ecosystems.

In answering the second research question–*what role do subjective experiences of aging play in incel identity formation*–the results highlight how the experience of growing older hardens the incel identity through a profound sense of temporal displacement and the active creation of an age-salient context. Feeling old is defined in the discussions by the acute awareness of being “off-time” relative to normative societal milestones (Stijelja and Mishara, 2023). Our analysis of retrospective accounts narrated by older incels for their peers reveals a complex and ambivalent affective landscape that challenges the monolithic view of incels as driven solely by active, youthful rage. While accumulated grievances occasionally manifest in violent fantasies, the emotional trajectory of the older incel is more prominently characterized by the coexistence of opposing emotions: resentment, anger, and grief blend with apathy, numbness, and emotional exhaustion. For some, aging stabilizes the certainty of permanent exclusion while simultaneously flattening the emotional energy required to sustain active anger. Some older users position themselves as bleak warnings of a permanent incel future, while others assume mentorship roles, actively encouraging younger members to persevere and insisting that the window of opportunity in youth is not yet closed, despite regarding their own fate as having been determined early in life.

The retrospective accounts analyzed in this article predominantly agree that adolescence is a critical phase of “arrested development.” Older incels frame teenage romance and sexual experimentation as essential developmental training necessary to meet rigid masculinity norms (see also Maryn et al., 2024). Missing these milestones is narrated not merely as a temporary deprivation, but as a permanent developmental deficit that leaves the adult self lacking the social and embodied capacities required to be a man. Incels treat specific ages as rigid “deadlines” or expiration dates after which self-improvement, normally facilitating romantic hope, arrives too late. In this age-salient context, aging is viewed not as a normal biological process, but is instead interpreted as empirical proof of final sexual disqualification and a perceived “expiration” of masculinity. The fact that masculinity is treated as an unattainable ideal, from which the older self is permanently disqualified, is a particularly salient result, because previous research shows that perceived failure to meet the Eurocentric hegemonic masculinity and the cisgenderist, heteronormative gender order associated with it serves as a core mechanism driving the ideological adherence of the forum (Aiolfi et al., 2024; Coufal et al., 2025; O’Hanlon et al., 2024). Research also shows (Coufal et al., 2025; Held, 2023) that rigid gender roles associated with a heteropatriarchal cisgenderist gender order are weaponized against outgroups of the incel ideology, which are women and people failing to adhere to the rigid gender roles, such as feminine men, masculine women, and individuals of non-heterosexual orientations.

Our results suggest that the same policing of gender order is applied to the self–that is, failure to acquire social experiences associated with hegemonic masculinity is narrated as a reason for loneliness and social exclusion, externalized misogynistic rage, grief, and apathy. This complex emotional state illustrates the affective mechanism of *ressentiment*, which politicizes loneliness and transforms isolation into misogynistic, antagonistic emotions (Tietjen and Tirkkonen, 2023). Through this discourse, genuine struggles with societal norms and social belonging are transformed through the lens of Eurocentric cis-heteronormative masculinity (Connell and Messerschmidt, 2005), patriarchal entitlement (Sugiura, 2021), and pseudoscientific theories of social order (Cannito and Ferrero Camoletto, 2022) into a rigid state of “wounded male victimhood” (Kelly et al., 2024). While this wounded male victimhood is intimately connected with violence (Díaz and Valji, 2019; Ferber and Kimmel, 2008), engaging with such toxic masculinity content also functions as a psychological trap, as it has been found to heighten depression, lower self-esteem, and worsen overall mental health among men (Parent et al., 2019).

Taken together, our results show how misogynistic ideology, loneliness, masculinity norms, and subjective aging can become woven into a life story in which exclusion is understood as permanent. This does not mean that aging produces a single emotional or ideological outcome. Rather, aging can intensify resentment, produce resignation, support claims to experiential authority, or create moments of ambivalent care toward younger users. Attending to older incels therefore broadens incel research beyond youth radicalization and shows how harmful online identities may be sustained, reinterpreted, and emotionally reorganized across the life course.

## Data Availability

The datasets presented in this article are not readily available. Requests to access the datasets should be directed to linda.coufal@fsv.cuni.cz.
